# Design and Modification of a Material Extrusion 3D Printer to Manufacture Functional Gradient PEEK Components

**DOI:** 10.3390/polym15183825

**Published:** 2023-09-19

**Authors:** Tobias Ritter, Eric McNiffe, Tom Higgins, Omid Sam-Daliri, Tomas Flanagan, Michael Walls, Pouyan Ghabezi, William Finnegan, Sinéad Mitchell, Noel M. Harrison

**Affiliations:** 1 School of Engineering, University of Galway, Galway, Irelandwilliam.finnegan@universityofgalway.ie (W.F.); sinead.mitchell@universityofgalway.ie (S.M.); noel.harrison@universityofgalway.ie (N.M.H.); 2 I-Form, the SFI Research Centre for Advanced Manufacturing, Ireland; 3 Éire Composites Teo, Údarás Industrial Estate, An Choill Rua, Inverin, Co., H91 Y923 Galway, Ireland; 4 CTL Tástáil Teo, Údarás Industrial Estate, An Choill Rua, Inverin, Co., H91 Y923 Galway, Ireland; 5 Construct Innovate & SFI MaREI Research Centre, University of Galway, Galway, Ireland; 6 Ryan Institute for Environmental, Marine and Energy Research, University of Galway, Galway, Ireland

**Keywords:** material extrusion printing, functionally graded materials, PEEK, fused deposition modelling, additive manufacturing

## Abstract

In recent years, the creative use of polymers has been expanded as the range of achievable material properties and options for manufacturing and post-processing continually grows. The main goal of this research was to design and develop a fully-functioning material extrusion additive manufacturing device with the capability to produce functionally graded high-temperature thermoplastic PEEK (polyether ether ketone) materials through the manipulation of microstructure during manufacturing. Five different strategies to control the chamber temperature and crystallinity were investigated, and concepts of thermal control were introduced to govern the crystallisation and cooling mechanics during the extrusion process. The interaction of individually deposited beads of material during the printing process was investigated using scanning electron microscopy to observe and quantify the porosity levels and interlayer bonding strength, which affect the quality of the final part. Functional testing of the printed parts was carried out to identify crystallinity, boundary layer adhesion, and mechanical behaviour. Furnace cooling and annealing were found to be the most effective methods, resulting in the highest crystallinity of the part. Finally, a functionally graded material cylindrical part was printed successfully, incorporating both low and high crystalline regions.

## 1. Introduction

The integration of additive manufacturing with traditional manufacturing techniques has brought about a transformative shift in the production landscape for polymers and composites. Throughout history, traditional manufacturing processes like hand lay-up, vacuum-assisted resin transfer moulding (VARTM), injection moulding, extrusion, and compression moulding have played a crucial role in fabricating polymers and composites on a large scale. However, with the advent of Industry 4.0, new methods have undergone significant advancements, enabling new product design possibilities and unprecedented efficiencies in production [1,2,3]. The traditional manufacturing methods mentioned above achieve high production rates, precise control over material properties, and cost-competitiveness. Additive manufacturing (AM, also known as 3D printing) has emerged as a valuable complement to these techniques. Additive manufacturing offers distinct advantages, such as the ability to fabricate complex geometries and customised designs with minimal bespoke tooling or material waste [4,5,6]. This makes it particularly suitable for prototyping, small-scale production, and rapid product development. By integrating additive manufacturing with traditional techniques, manufacturers can leverage the strengths of both approaches, resulting in greater flexibility, improved efficiency, and enhanced product innovation within the realm of polymers and composites.

Advances in AM technologies in recent years have led to faster design and prototyping phases in new product development and the emergence of new advanced product and tooling designs. In addition, 3D printers have become increasingly affordable as the market has evolved from to span research, industrial and consumer applications. Material extrusion (MEX) additive manufacturing (of which fused deposition modelling is a common subset) is the most readily attainable 3D printing technology available. Additive manufacturing is particularly suited to the production of functionally graded materials (FGMs). This type of component, in which the structure, chemistry, or microstructure and thus properties are intentionally varied within a single part, is the next frontier in manufacturing technology, promising a broad range of applications across many industries, including medical devices and aerospace [7]. In plastics, FGMs require a semi-crystalline polymer in order to vary the material properties within the extremes of maximum and minimum achievable crystallinity. This rules out the most common MEX printing materials of ABS and nylon, as they are amorphous polymers. PLA is a common MEX material and is a semi-crystalline polymer; however, its level of crystallinity is low, and the temperature range in which its crystallinity can be altered is small, which also renders it unsuitable for a MEX FGM demonstrator. PEEK (polyether ether ketone) exhibits high crystallinity and a wide temperature range in which its material properties can be altered. A semi-crystalline polymer will crystallise when cooling from its melted state or from being heated from an amorphous solid state [8]. 

The benefit of using MEX as a manufacturing process to realise functionally graded parts is that it is a gradual process in which neighbouring regions of the part are manufactured in sequence. When compared with traditional near-net-shape manufacturing methods, such as casting or moulding (where the part is fully manufactured as one), MEX offers much more local control of the material during manufacturing. Thus MEX allows dynamic tuning of the print process parameters to vary the thermal conditions required for microstructure control. PEEK is a challenging material to extrude and print in homogenous form due to the higher melt temperature, and therefore, developing a capability for functionally graded material extrusion involves many challenges in the design and optimisation phases. 

The primary MEX production parameters, including temperature, speed, nozzle diameter, layer thickness, and filling ratio, affect the strength of the produced part [9,10,11,12]. The temperature settings (nozzle, build plate, and ambient) can be regarded as the most imperative to success due to their direct influence on the flow characteristics and the crystallisation process. Direct ink writing (DIW) 3D printing is a prominent technique for thermally cured thermosets, akin to MEX. Unlike MEX, DIW employs pressurisation instead of heat to lower viscosity. Feedstock from pressurised syringes is deposited fluidly, often at room temperature, eliminating the need for melting [13,14,15]. Honglei et al. [16] examined 3D-printed PEEK parts in their research, studying the effects of the extrusion rate, filling angle, and orientation on mechanical properties. Optimal results were achieved at 1.0× extrusion rate, variable ±10° angle fillings, and vertical printing, with heat treatment at 300 °C for 2 h significantly improving crystallinity and strength to nearly 80% of the injection-moulded parts’ strengths. Paridokht et al. [17] explored how mechanical properties are influenced by printing parameters like orientation and position. Using PEEK filament and the MEX process, some samples were printed and categorised on the basis of print orientation, with the mechanical analysis showing a print orientation dependency.

In 2016, NASA published a paper about their efforts to convert an off-the-shelf, low-cost 3D printer into a high-temperature printer with PEEK printing capability [18]. They used a Lulzbot Taz 3D printer, and the team made similar upgrades to their printer, including a thermal enclosure, a drying chamber for the filament, a hot end upgrade, motor cooling, and the replacement of some plastic components with more temperature-resistant materials. The team built the printer to print PEEK without the capability of FGM. They successfully printed PEEK parts, having overcome some of the major challenges of printing with the material, such as the high temperature required and the need for ambient temperature control. Temperature control of the surroundings is important since a large temperature gradient would cause the filament material to warp and deform during the build process. Higher ambient temperature also improves inter-laminar adhesion between the printed layers. PEEK is also a hygroscopic material, which can cause porosity issues within the print, causing inconsistencies that are undesirable for high-performance parts. This was resolved by drying the material first using an oven and a drying agent. 

Yang et. al achieved success with 3D printing functionally graded PEEK parts through MEX by controlling the nozzle and ambient temperature and heat treatment methods (oven annealing) to control the crystalline structure of the material [19]. PEEK exhibits high strength and biocompatibility, which make it a candidate material for orthopaedic medical implants [20]. It was found that the crystallinity of PEEK could be controlled through three main measures using MEX: ambient temperature control, nozzle temperature, and post-processing treatments [19]. By introducing 10 mol% fluorene groups into the polymer chain (10%-FD-PEEK), the interlayer strength of 10%-FD-PEEK could be improved to 41 MPa, which is close to that of the commercial PEEK resin for 3D printing (Victrex, AM 200) [21]. The European Space Agency (ESA) has been developing a strong connection with PEEK since 2008 [22], and as part of their continued interest, they have realised the new potential for this material. During space missions, crews often require new parts due to failure or damage. This can range from seemingly rudimentary applications, such as crew toothbrush handles, to mission-critical components (e.g., liquid transport pipes) [23]. Combining this extremely inaccessible space environment with a distinct lack of resources or spare parts can mean a real impairment for a mission. As every kilogram of cargo sent into space costs upwards of EUR 78,500 [24], there are obvious benefits to reducing spacecraft payloads. There have been recent breakthroughs in additive manufacturing of PEEK by private companies, such as Apium [25] and Markforged [26], and separately, NASA [18] and researchers in China [19]. This research has shown that it is possible to manufacture high-quality PEEK parts by additive manufacturing and not just through machining and injection moulding, opening the door for a broader range of parts to be developed as well as PEEK as an FGM. 

The main aim of this work was to design, assemble, and test a cost-effective MEX 3D printer capable of printing high-quality functionally gradient components from PEEK. This involved the optimisation of the printing parameters for PEEK and verification that dynamic control of the process parameters leads to the fabrication of an FGM part. One of the biggest factors limiting a wider update of PEEK 3D printing is the limited availability of affordable high-temperature PEEK printers, and thus a specific objective was to minimise hardware cost.

## 2. Conceptual Design

**Figure 1:**
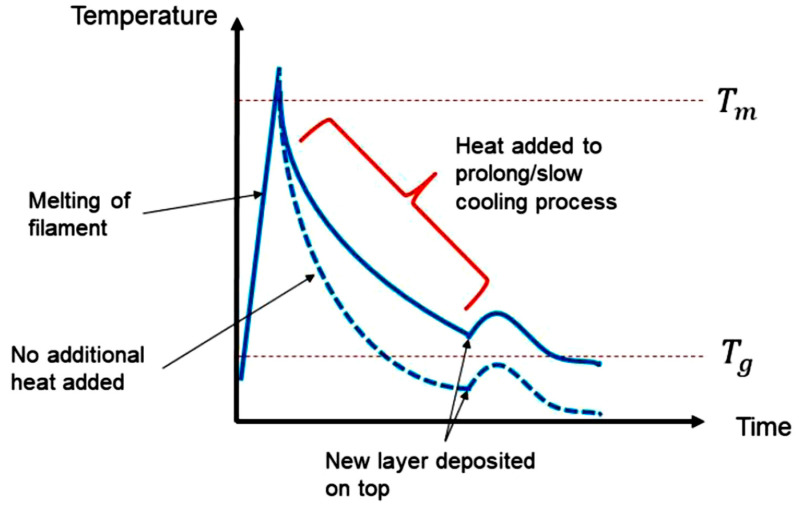
Typical temperature–time curve during a MEX process [27].

The main objective was to produce parts with a functional gradient through the control of the component cooling and crystalline microstructure evolution. The fundamental method in which this is carried out is by adding heat to the part after printing to reduce the overall cooling rate so that higher crystallinities may be achieved as the material cools (Figure 1).

**Figure 2:**
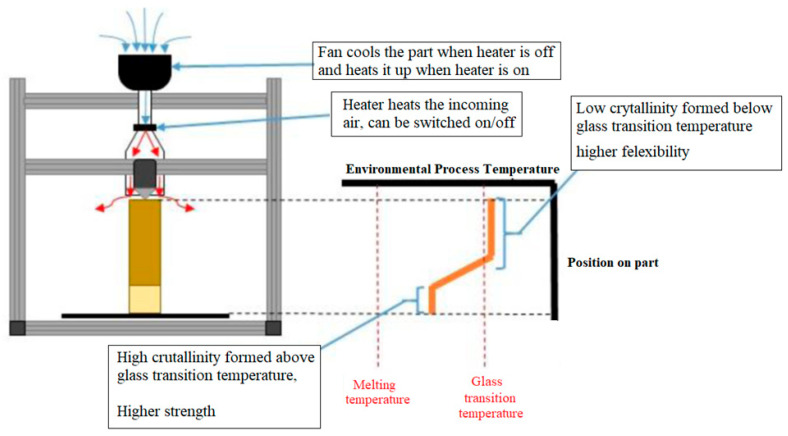
Hot air delivery concept.

**Figure 3:**
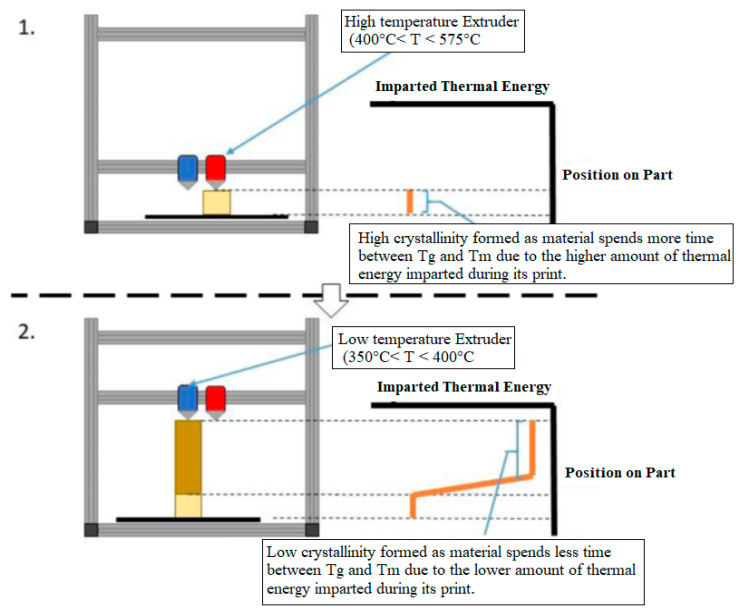
Dual nozzle extruder concept, 1. High temperature extruder, 2. Low temperature extruder.

**Figure 4:**
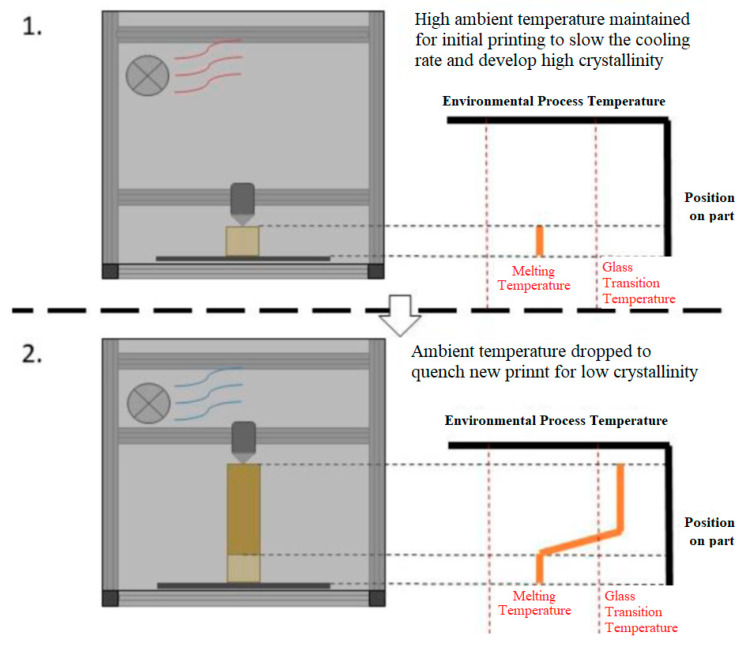
Ambient temperature control concept, 1. High ambient temperature, 2. Low ambient temperature.

**Figure 5:**
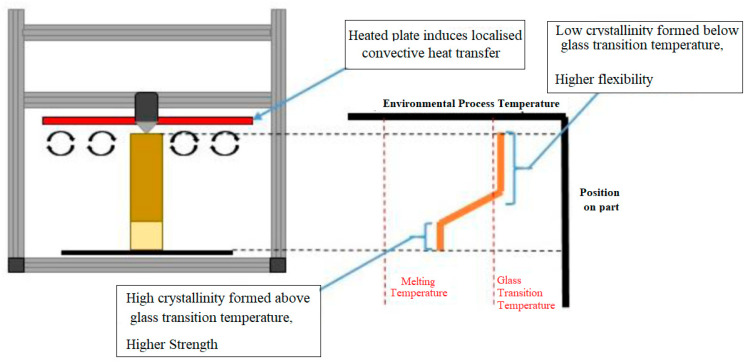
Concept schematic of supplementary heating.

**Figure 6:**
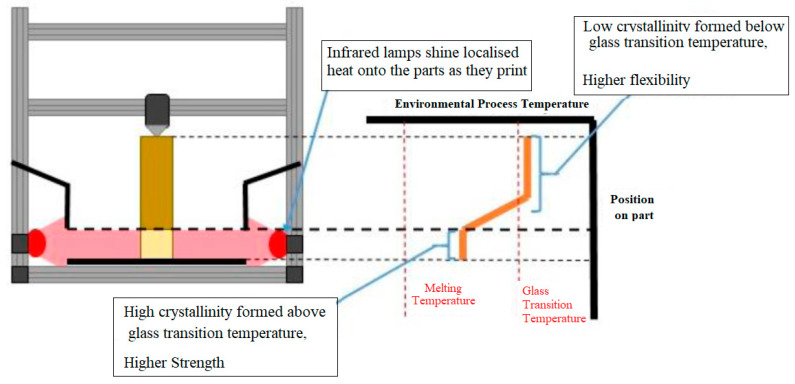
Infrared lamp concept.

Ensuring a sufficiently high temperature in the previous layer is crucial for facilitating cross-layer crystal and chain formation in conjunction with the new layer, ultimately enhancing the structural integrity and uniformity of the final part. Within this section, various concepts are presented, each offering distinct methods and approaches for introducing additional heat energy into the material, as illustrated in Figure 1. Standard MEX printers lack the capability to vary the thermal processing conditions throughout the printing process. Consequently, to achieve this capability, it becomes necessary to incorporate one or more of the proposed concepts: hot air delivery, dual nozzle design, ambient (chamber) temperature control and enclosure, heated plate, or supplementary infrared heaters. The details of each concept are presented in Figure 2, Figure 3, Figure 4, Figure 5 and Figure 6, with more info provided in [28].

### 2.1. Concept 1: Hot Air Delivery

An inline heater is present in the air ducting, which can be turned on and off during the printing, as shown in Figure 2. With the heater turned off, the fan alone can “quench” the material, causing it to have a small percentage of crystallinity. With the heater turned on, the hot air ensures that the newly deposited material spends as much time as possible above the glass transition temperature, allowing the maximum amount of crystallisation to occur.

### 2.2. Concept 2: Dual Extruder

This proposal outlines a method of achieving functional gradient by using a “hot” and “cold” nozzle to print high crystalline and low crystalline sections. 

Both would run in parallel, starting and stopping when indicated by the user for desired properties. Software support for splitting up a print into two materials currently exists. A Chimera+ extruder head was designed to print the main material as well as a support material, which is typically weaker so that it can be easily removed after printing. 

### 2.3. Concept 3: Ambient Temperature Control

This design controls the ambient air temperature through a heating element and a fan, much like a conventional oven. A schematic diagram for this concept is presented in Figure 4.

### 2.4. Concept 4: Supplementary Heating

A potentially viable external heat source design to control the cooling rate of the part is a circular hot plate design located around the extruder head (Figure 5). The idea behind this concept is that the plate would be heated throughout the print to the desired temperature, heating the layers that have already been placed by convection and thus maintaining the part at a steady temperature, slowing down cooling. 

### 2.5. Concept 5: Infrared Lamp Supplementary Heating

This concept uses concentrated infrared lamps to maintain a high temperature on a specific part of the piece while it is printing (Figure 6). This allows a higher crystallinity to be developed in the section the lamp is shining on. 

In order to identify the most suitable concept for the final design, a decision matrix was developed. This matrix assigned scores to various concepts that were proposed, ultimately leading to the selection of the optimal idea. Among the evaluated options, the application of heated plates (concept 4) and infrared bulbs (concept 5) for targeted heat distribution received the highest score. The final design incorporated valuable insights gained from research and conceptualisation, resulting in a hybrid solution. This approach combined the use of infrared heaters in a manner similar to the heated plate, successfully integrating the best features from both concepts.

## 3. FGM MEX Printer Design

### 3.1. Development and Modifications

The concept of using a prefabricated 3D printer kit is a largely accepted and well-developed method for industrial and educational purposes in the science of additive manufacturing. A typical starter kit can cost in the range of EUR 400–1000 and is available from many online retailers. The device chosen (Velleman K8200 kit, Velleman, Ghent, Belgium) was supplied by Velleman and requires full assembly, including all mechanical, electronics, and interfacing, to be carried out by the customer. Thereafter, the next step was to modify this kit to extrude the PEEK filament and subsequently develop PEEK FGM capabilities. 

Build limit dimensions dictate the overall maximum geometric dimensions of printed parts. The minimum requirement was judged to be at least the required dimensions of a standard mechanical test specimen. The (minimum) ASTM D638 standard dimensions for a standard tensile test specimen are 63.5 × 9.53 × 3.2 mm (L × W × H). Larger build dimensions are favourable, as this allows for a larger range of parts to be manufactured. The build envelope for the Velleman K8200 purchased for modification is 200 × 200 × 200 mm.

Material feed is important for the overall design specification, as this dictates the speed at which filament layers can be extruded onto the print bed. High speed is not always advantageous, as this can lead to inaccurate prints. Printer head movement for the Velleman K8200 was restricted to movement along the *z*-axis, whereas the print bed could move along the x- and y-axis. The extruder being fixed to only one axis has some inherent advantages, including the retention of more available space for ancillary heating or sensing devices, if needed. In order to withstand the higher ambient temperatures associated with PEEK printing, the standard motor and drive assemblies of a Velleman K8200 needed to be either isolated from the enclosure, replaced with temperature-resistant components, or protected with cooling shrouds. This also requires plastic mounting pieces to be replaced, as well as pulley belts. 

Since the frame of the Velleman K8200 is already made from extruded aluminium, it permits relatively easy customisation and offers a range of mounting points for components. The ambient temperature in the enclosure has to be maintained in the range of the glass transition temperature (Tg) and the melting temperature (Tm) of PEEK (145–375 °C). This prevents the warping of the printed part due to the temperature gradient between the extruded polymer and the air temperature and improves layer fusion. Polycarbonate sheeting was used as a low-cost enclosure in combination with the aluminium extrusion. Finally, since PEEK is a hygroscopic material, it has to be stored and dried in an enclosure before use in a MEX process. Drying can be achieved using silica gel, desiccant, or similar drying agents.

**Figure 7:**
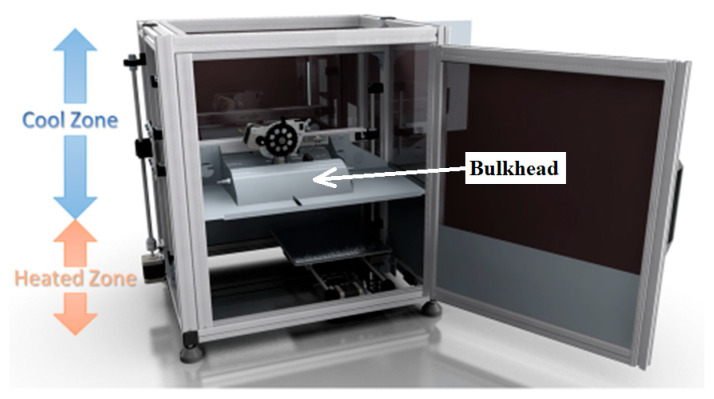
CAD model showing internal modifications and heated/cool zones.

The functionality of the kit’s standard format allows for the extrusion of low-performance, low-melting-point polymers, such as ABS and PLA. To facilitate extrusion and FGM of PEEK, the purchased kit required extensive modification. CAD and FEA models were used as tools to develop the final design solution. The full CAD model, as pictured in Figure 7, for the standard stock model of the Velleman K8200 was downloaded and cross-checked for dimensional accuracy against the actual machine. At the initial design stages, CAD models of upgraded components were assembled onto the Velleman K8200 CAD model, saving time and allowing for informed design decisions to be made. The final designed model was then iteratively produced in CAD to reflect the end product, as per Figure 7.

Functional testing of the printed parts was carried out to identify the formation of crystallinity, boundary layer adhesion, and mechanical behaviour. The behaviour of the extruded parts in the tests outlined above provided feedback for functional optimisation of the extrusion process, as detailed in [28]. The final prototype was designed so that all variables of the print parameters could be tightly controlled so that they could be refined through continued optimisation.

### 3.2. Manufacturing and Assembly

To reduce costs, the number of parts was minimised while also reducing the time taken to design custom components. A reduced quantity of connections led to fewer fixtures required and increased reliability. Wherever possible, lightweight sheet metal connection techniques were used, such as soldering and rivets. Sheet metal parts formed a large volume of the parts required for fabrication, such as the bulkhead, which separates the high-temperature volume from the “cooler” volume. The bulkhead part was formed as shown in Figure 7 with folded edges. The folded edges were used as a weight-saving stiffening technique, as the bulkhead was suspended from the *z*-axis carriage. 

Zinc-coated (6 microns) steel sheets with a thickness of 0.2 mm were used, thus ensuring that oxidisation issues would not be a concern. Any exposed edges that were drilled or cut were refinished using a zinc base cold galvanising spray paint, thus safeguarding the longevity of the part’s service lifetime. This protection was applied to all parts to guarantee that a high-quality rig would be produced. The aforementioned 0.2 mm tinned steel sheets were supplied in a 500 × 300 mm sheet size, and because the bulkhead’s overall dimensions were larger than this, a rivet joining method was used. 

Wherever possible, standard parts were integrated into the design, such as commercially available 90-degree brackets, connection plates, standard nuts/bolts, and aluminium profiles. As can be seen in Figure 7, a bulkhead was designed to separate the heated lower zone from the cooler upper zone. This design had the effect of decreasing the heated volume, leading to increased energy efficiencies and eliminating the need to replace all the parts above the hot end to manage the new higher operating temperatures. This part was designed to be lightweight while providing a heat shielding effect from the hot end to the extruder assembly, thus protecting the sensitive motor, wiring, fans, and extruder ABS parts from overheating. 

As part of the PEEK extrusion and FGM process, a localised heat source is required to thermally affect the printed parts [19,29]. Infrared lights were chosen to perform this function on the basis of a number of advantageous factors. Infrared lights are an easily controlled heat source compared with convection or conduction methods, minimising heat loss to the surrounding environment through convection currents, which can occur from a heater cartridge-style setup similar to Apium’s heated plate. Due to the lightweight nature of the infrared bulbs (30 g each), they were able to be attached to the *z*-axis arm without the need to increase the power output for the *z*-axis motor. 

**Figure 8:**
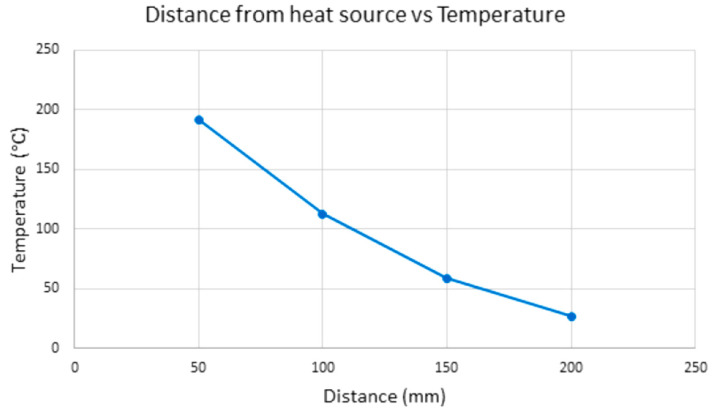
Distance vs. temperature reached in the infrared heating elements.

By having the thermal heat source fixed to the *z*-axis, the heat energy applied to the extruded parts to induce functional gradient crystallinity levels can be controlled on a layer-by-layer basis or per group of layers simultaneously. The cooling rate can also be controlled using this heat source. As the interlayer adhesion and temperature differentials in the parts can lead to mechanical defects, such as the loss of stiffness and warping, respectively [29], acute control of the extrusion process is required. Infrared Quartz elements were chosen as the infrared source due to their relatively small dimensions in comparison with the rig. An experiment was set up to test the effects of a 1000 W Quartz heating element on a sample of PLA. A part previously printed on the standard K8200 rig was used for this purpose. The graph in Figure 8 shows the temperatures reached on the PLA part at specific distances from the infrared heater element using a hand-held thermal sensor at a time period of five minutes to allow for sufficient heat absorption to occur in the part. It was found that the maximum temperature reached at 50 mm from the infrared element was 192 °C after five minutes of heating time. The temperature reached was deemed sufficient, as this was above the glass transition temperature for PEEK.

**Figure 9:**
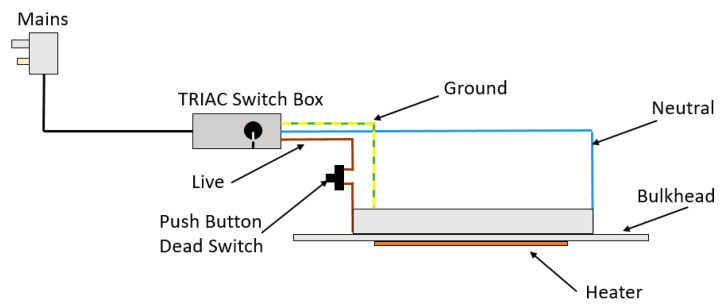
The heater implementation with safety measures and temperature control.

A dead switch was wired into the circuit as a safety precaution, which was then mounted on the door frame so that the heater could not turn on while the door was open, preventing users from touching the hot elements. Secondly, a TRIAC switch was also wired into the circuit. This allowed for varying the supply voltage to the heater and thus reducing/increasing the temperature as required to apply different settings of heat treatments, essentially acting as a dimmer switch, which is commonly used in lighting applications. For safety, the TRIAC switch was mounted to the outside of the frame inside a custom 3D-printed enclosure. The schematic for the heater integration circuitry is shown in Figure 9.

To successfully melt PEEK at the printer hot end, a temperature of approximately 400 °C is required, as the melting point of PEEK is 343 °C. To remove all existing semi-crystalline regions and ensure that a fully amorphous microstructure is created in the melt, a temperature above Tg is required [25]. It is worth noting that recrystallisation starts at the crystallisation temperature range (170 °C and 310 °C) upon cooling [30]. The stock hot end heater cartridge on the K8200 can reach a maximum operating temperature of 190 °C. The stock hot end was replaced with an all-metal “V6 hot end” from E3D (Oxfordshire, UK). This aluminium hot end is capable of handling temperatures of over 400 °C in combination with a PT100 temperature sensor. Due to the expected heat flow through the new aluminium hot end to the extruder assembly, cooling features were added to the hot end, even though the hot end already featured a heat sink. A cooling fan was installed and pointed directly at the hot end heat sink to increase the cooling effect of air flowing over its surface area. To limit the effects of thermal bridging, a Nylon PA12 (Tg range = 80–190 °C) heat break of extremely low thermal conductivity (0.231 W/(m·K)) was used to couple the hot end to the extruder. This new hot end also featured insulated cables to ensure heat shielding against the new higher operating temperatures. 

To raise the temperature of the PEEK inside the hot end to temperatures above its melting point, a heater cartridge inside a plated copper block was chosen. Copper, a suitable material, exhibits a high melting temperature (Tm = 1085 °C) and a high thermal conductivity of 401 W/(m·K), thus allowing for effective heat transfer to take place between the heater cartridge and the PEEK filament. This copper block was nickel-plated, which significantly reduces the adhesion of PEEK to the block due to its low coefficient of friction.

**Figure 10:**
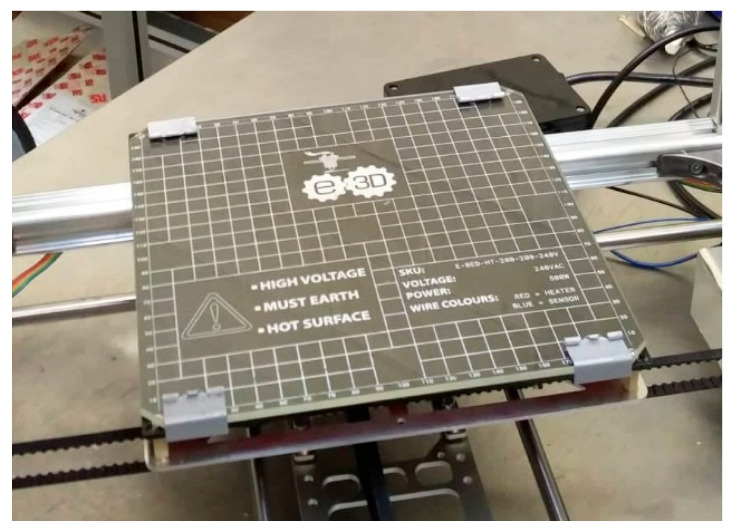
Heated bed fitted to the FHM MEX machine.

**Figure 11:**
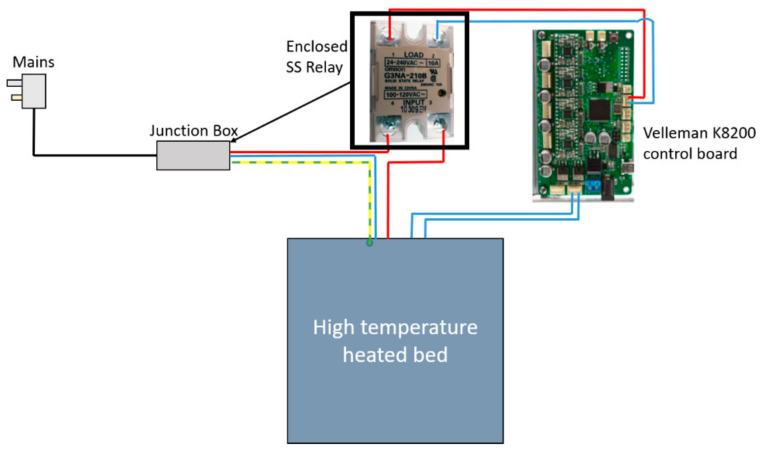
Schematic diagram of wiring setup for the heater bed upgrade.

At the tip of the hot end, the melted PEEK was extruded through a heated nozzle. The internal geometry, material, and diameter of the opening on this nozzle play a major role in a successful extrusion process. A 0.4 mm diameter nozzle manufactured from hardened steel was also purchased and fitted to the hot end to facilitate the extrusion of highly abrasive materials, such as electronically conductive carbon-doped PEEK. The hardened steel nozzle shows the high wear properties required for this demanding application. In order to avoid steep temperature differentials and the associated detrimental effects, a printing bed with an operating temperature closer to the extruded PEEK temperature (approx. 400 °C) was required. Concurrently, the temperature of the bed must facilitate printing at higher temperatures to assist with crystallinity growth at a temperature capability above PEEK’s glass transition temperature (Tg = 143 °C). The stock K8200 hot bed was capable of a maximum operating temperature of 60 °C; therefore, a high-temperature 500 W bed with a maximum operating temperature of 200 °C was procured and fitted to the existing *x*-axis carriage, as per Figure 10. Since the new bed upgrade had significantly higher power requirements than the stock print bed, the bed could not be connected directly to the board to draw power. The bed had an integrated thermistor, which could be wired directly into the board to inform it of the current temperature of the bed. The layout of the circuit can be seen in Figure 11.

**Figure 12:**
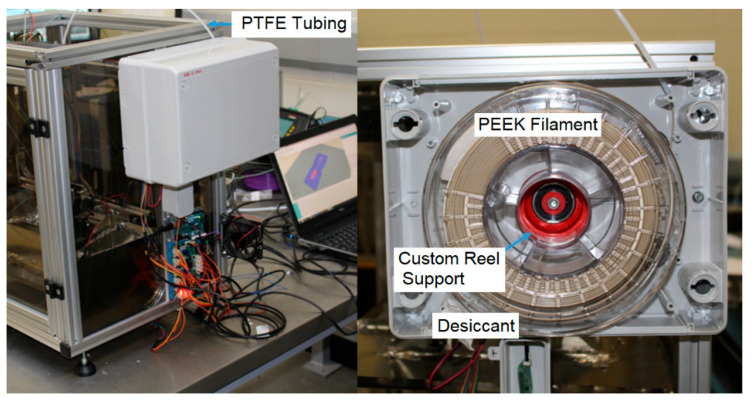
Filament enclosure.

To reduce the energy required to control the process temperatures, a reduced volume was accomplished by means of creating an enclosure for the rig. Clear polycarbonate (Tg = 147 °C) sheets with a thickness of 6 mm were used to enclose the rig. As hot air is generated by the process, this enclosure was also necessary for health and safety purposes. It was found that companies such as Intamsys, whose Funmat HT printer retails at approx. EUR 5500.00, produce similar MEX printers engineered to include an ambient process temperature of only 90 °C when extruding PEEK. In these cases, the machines are a seemingly low-cost solution but require a post-processing stage because an ambient temperature of 90 °C is too low to achieve high crystallinity levels, thus reducing the overall mechanical performance of the printed component. In order to avoid a two-stage processing setup and to ultimately possess the ability to control crystallinity levels at specific areas in a part, a high-temperature enclosure was required. An electrical junction box with an IP65 rating was purchased and converted into a filament storage dry box. The filament box can be seen in Figure 12. The red component is the custom reel mount and the PTFE tube is also highlighted.

**Figure 13:**
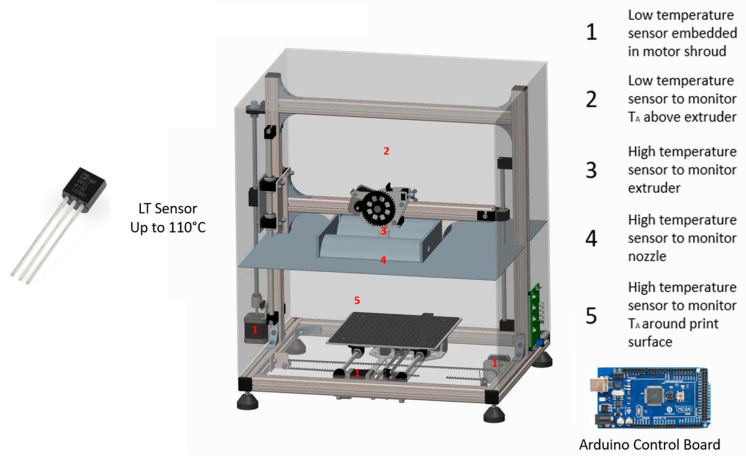
Motor temperature control.

**Figure 14:**
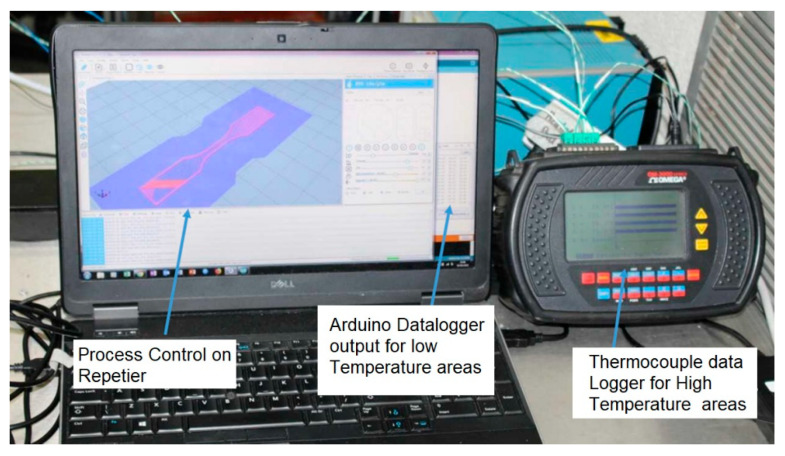
Temperature and process control during printing.

Electric motors can be particularly sensitive to thermal degradation, which can lead to missed steps; this ultimately leads to premature failure and additional costs. There are four stepper motors installed on the rig to provide linear x, y, and z movement and feed the PEEK filament through the hot end. The motors driving the *x*- and *y*-axis, which would be subjected to high temperatures in the heated zone, were heat-shielded in a cooling shroud. To reduce the likelihood of motor failure, an Arduino-based temperature sensor data logger was built, wherein TMP37 temperature sensors were soldered to a shielded wire and attached to the sides of the motors, as shown in the schematic in Figure 13 below. For the high-temperature areas, such as near the heater, extruder, and print bed, a thermocouple data logger was used (Figure 14).

## 4. Optimisation of 3D Printing 

Many test prints were carried out using PLA to set up all the variables in the printing process before moving on to printing PEEK. Specimens were then printed from Luvocom^®^ 3F PEEK filament (Table 1) and tested before moving on to inducing the variation of crystallinity levels in parts. 

The process of optimising the printing of PEEK material was an intricate endeavour that hinged upon the strategic manipulation of essential process parameters, each wielding a discernible influence on the final product quality. These parameters were orchestrated with a keen focus on regulating the ambient temperature to facilitate precise material deposition and subsequent solidification.

Central to this optimisation effort were the crucial variables of infrared temperatures and heated bed temperatures. The former encompassed a meticulously calibrated range spanning from 20 °C to 140 °C, with a tight allowable variance of +5 °C/−5 °C. Similarly, the latter parameter, the heated bed temperature, was deftly managed within a controlled spectrum of 40 °C to 150 °C, maintaining the same permissible tolerance limits of +5 °C/−5 °C. These temperature settings were meticulously chosen to strike a delicate balance between ensuring optimal material flow and adhesion and avoiding any undesirable effects associated with overheating or cooling.

**Figure 15:**
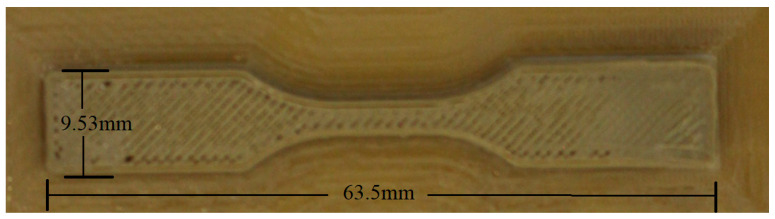
Printed PEEK dog bone sample with printed brim.

In tandem with temperature control, meticulous attention was dedicated to fine-tuning other pivotal printing parameters. Print speeds, varying from 15 mm/min to 60 mm/min, were adroitly adjusted to govern the localised temperature conditions and influence material deposition behaviour. The selection of specific infill patterns further contributed to the attainment of desired structural integrity and mechanical properties in the final printed objects. In preparation for each printing operation, meticulous steps were taken to ensure the favourable adhesion of the PEEK material to the heated bed’s glass surface. This crucial phase involved the application of Pritt stick, a commercially available adhesive. By creating a slightly textured surface on the glass, this adhesive played a pivotal role in enhancing bed adhesion. This effect was particularly pronounced when coupled with the implementation of a wide brim, which facilitated a secure foundation for the filament during the printing process. An array of bed preparation techniques, including PEEK film, hot glue, high-temperature tape, and masking tape, were exhaustively explored in pursuit of the optimal adhesion solution. The innovative brim feature, consisting of a single-layer thick perimeter encircling the printed object, emerged as a valuable asset. For instance, the dog bone specimen exhibited an optimal offset brim thickness of 12 mm, which strikingly balanced the demands of ensuring robust bed adhesion while curbing material wastage (Figure 15).

This approach enabled the accomplishment of high-temperature printing. For printing at lower temperatures in the range of 65–100 °C, a standard stationary adhesive (Pritt stick) was applied to the build plate, along with a specified brim on the first layer. In order to print at very low temperatures of approximately 20–65 °C, a technique involving the use of PEEK film was implemented. The film was secured to the bed using high-temperature tape. The utilisation of PEEK film aided in achieving bed adhesion, considering the challenges associated with printing PEEK at temperatures below 65 °C. The most critical process variables, which were identified through empirical observations, are outlined in Table 2. These variables represent the optimal printing conditions for promoting high crystallinity formation while ensuring satisfactory working conditions, minimal distortion, and sufficient bed adhesion.

A number of modifications were made to the stock printer at both the physical and firmware levels. To incorporate the high-temperature hot end into the prototype effectively, the firmware was tuned using a temperature vs. output voltage table in a configuration table. The Repetier 2.3.1 software, which served as the initial foundation for controlling the print parameters, proved to be inadequate for achieving the level of precise process control necessary. Consequently, the slicing software Ultimaker Cura 4.3 was employed as an alternative. Cura allowed for greater customisation by enabling the specification of parameters such as the brim width and individual print speeds for the perimeter and infill. This transition to Cura addressed the need for tighter process control in printing operations.

## 5. FGM Printing Results and Discussion

The tensile tests were meticulously conducted within controlled environmental conditions, maintaining a humidity level of 50% and a temperature of 23 °C. These specific parameters were chosen to ensure consistent and reproducible test conditions, thus enhancing the reliability of the subsequent analyses.

The conditions that governed the printing of the PEEK samples, as well as the resulting tensile strength outcomes for the different samples, are thoughtfully documented in Table 3. All samples conformed to established standards and were meticulously manufactured in a flat format, showcasing minimal instances of warping. Moreover, these samples demonstrated a commendable adherence to the specified dimensions, well within the tolerance limits defined by the standard. The initial samples denoted A1 through A6 encountered notable degrees of warping, a challenge that was effectively addressed in subsequent iterations. Dedicated efforts were undertaken to mitigate the ingress of external airflow from the cooling fan into the build chamber, a strategy that effectively minimised thermal gradients and the associated residual stresses. This successful mitigation led to a remarkable reduction in warping issues, signifying an incremental improvement in the manufacturing process.

For the samples labelled B1 through B6, the printing environment was maintained at an ambient temperature of 120 °C. Remarkably, the ultimate tensile strength (UTS) outcomes across this range displayed admirable consistency, ranging from 43.4 to 49.87 MPa. This minimal variation not only underscores the repeatability in the quality of the printed samples but also affirms the stability of the manufacturing process at this temperature setting. The final set of tensile specimens, designated as C1 through C4, were fabricated within an ambient enclosure temperature of approximately 140 °C. It is noteworthy that within this set of data, there were two outliers, exemplified by samples C1 and C3. By contrast, samples C2 and C4 closely mirrored the strengths exhibited by the specimens manufactured at the lower temperature of 120 °C. The deviation in the two outliers can plausibly be attributed to minor manufacturing imperfections, particularly in the form of slight warping. This slight distortion was amplified during the loading process in the jaw clamps of the tensile test rig, ultimately leading to premature cracking and a reduction in the final failure load.

**Figure 16:**
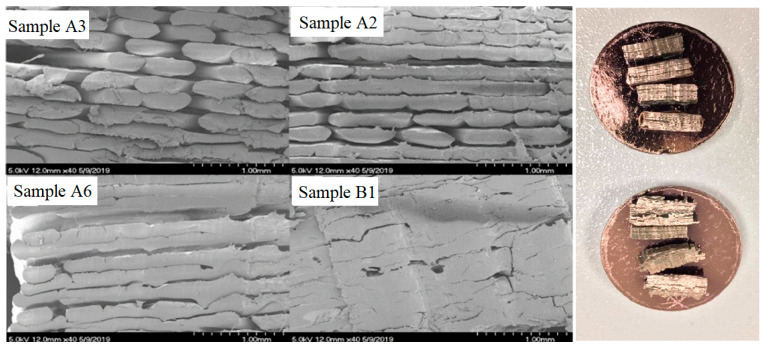
Scanning electron microscopy (SEM) testing results [28].

Scanning electron microscopy (SEM) was carried out using a Hitachi S-4700 Scanning Electron Microscope (Tokyo, Japan) on extruded PEEK parts with the purpose of characterising the microstructural formations (Figure 16).

The scanning electron microscope (SEM) analysis of the samples offers a compelling visual insight into the transformative impact of the fabrication process. Notably, the observed porosity levels exhibited a remarkable reduction, portraying the evolution from an initial porosity level of 39.5% in sample A3 to an impressive reduction of 4.2% in sample B1. This trend of porosity reduction was similarly reflected in the results from samples A2 (initial porosity of 17.4%) and A6 (initial porosity of 9%), highlighting the consistent effectiveness of the manufacturing process in addressing this crucial aspect.

The rationale behind employing SEM testing was grounded in the necessity to meticulously validate that the implementation of functionally graded materials (FGMs) does not give rise to any compromises in the mechanical properties of the fabricated components, nor does it inadvertently compromise the interlayer adhesion. By closely examining the microstructure through SEM, any potential deviations, irregularities, or shortcomings in terms of porosity and interlayer bonding can be definitively assessed. This analytical rigour underscores the commitment to ensuring that FGM integration occurs seamlessly and contributes positively to the overall mechanical integrity and performance of the produced components.

To thoroughly investigate the extent of crystallinity within the printed components, a systematic examination was conducted involving the assessment of two distinct categories of parts: one exhibiting low crystallinity and the other characterised by high crystallinity. This meticulous analysis was undertaken to elucidate the intricate nuances of crystalline structure distribution within the fabricated objects. To ensure methodological rigour, the fabrication of these test specimens adhered precisely to the prescribed process parameters detailed in Table 4. This stringent adherence to predefined manufacturing conditions ensured consistency and reliability in the ensuing assessment.

**Figure 17:**
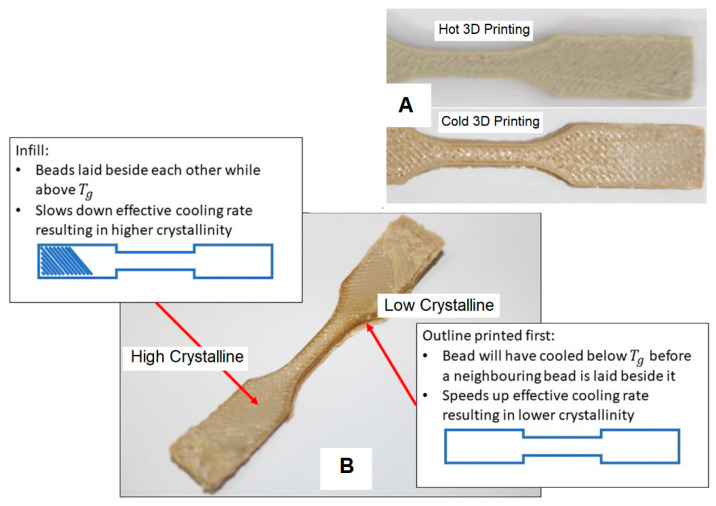
Colour variation between the “Cold” print and the “Hot” print, (**A**) Samples printed with hot and cold printing conditions, and (**B**) Different crystalline levels in a 3D printed specimen [28].

The outcomes of this deliberate investigation are visually documented and effectively communicated through the graphical representation provided in Figure 17. The visual depictions within this illustration serve as a poignant visual record of the transformation in colouration, which inherently serves as a reliable indicator of the evolving crystallinity status within the printed components.

Significantly, the colour shifts observed in Figure 17 serve as compelling markers that signify the advancement of crystallinity growth within the fabricated structures. The juxtaposition of distinct colour tones distinctly outlines the boundaries between varying degrees of crystallinity. This not only underscores the responsive nature of the 3D printing process to thermal dynamics but also underscores the fundamental role played by temperature conditions in shaping the material’s structural attributes.

Ultimately, this comprehensive analysis contributes valuable insights into the intricate relationship between the crystalline characteristics of printed components and the thermal conditions encountered during both “cold” and “hot” 3D printing scenarios. This deeper understanding of crystallinity behaviour enriches our comprehension of additive manufacturing processes, thus paving the way for enhanced material manipulation and optimisation.

In comparison with prints exhibiting full amorphous characteristics, it can be anticipated that a low crystalline print would manifest a significantly deeper and more translucent colouration. This pronounced disparity is vividly presented in Figure 17. The left segment of Figure 17 (labelled as the “high crystalline zone”) showcases a printed component subjected to a fan-directed cooling approach, thereby enhancing the cooling process. Conversely, the right section of Figure 17 (referred to as the “low crystalline zone”) depicts an alternative print test specimen characterised by a colder printing environment.

It is evident in the visual representation in Figure 17 that the “cold” test specimens did not attain a complete amorphous structure. The underlying cause for the observed divergence in crystallinity can be attributed to the printer’s infill pattern, which introduced sufficient heat to crystallise the central region of the part effectively. However, this crystallisation phenomenon was not as pronounced when the printer began laying down the border at the initiation of each layer.

**Figure 18:**
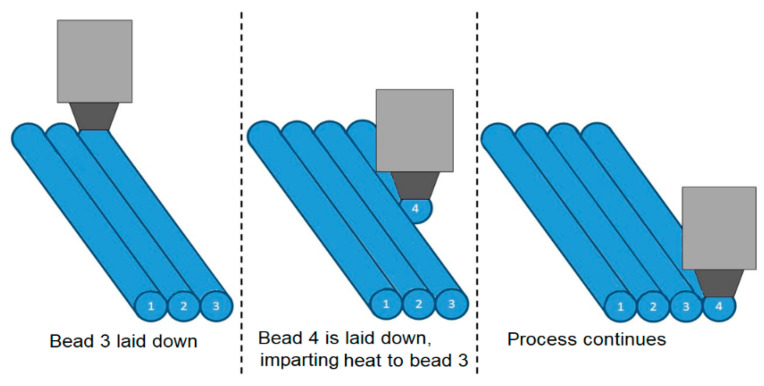
Effect of printing process on thermal history of a zone.

Furthermore, the close proximity of the hot end to the adjacent beads during their placement might exert a substantial influence on the cooling rate. This intricate interaction is elucidated in Figure 18, which delineates how the printing process introduced additional heat to a bead, thereby inducing a deceleration in the cooling progression for that specific part. To delve deeper into the implications of the infill pattern and the proximity of the hot end to the component, a thermal imaging camera was employed to meticulously observe the unfolding of the printing process.

## 6. Conclusions

This study developed a functional additive manufacturing device capable of producing functionally gradient high-temperature thermoplastic PEEK materials by manipulating their microstructure during the manufacturing process. Different strategies were offered for controlling chamber temperature and crystallinity while introducing thermal control concepts to govern the extrusion process’s crystallisation and cooling mechanisms. The achievement of this work can be summarised as follows:

- Five different strategies to control the chamber temperature and the crystallinity were illustrated and compared: hot air delivery, dual nozzle design, ambient temperature control and enclosure, heated plate, and supplementary infrared heaters. The final design was a hybrid option wherein infrared heaters were used in a similar fashion to the heated plate.
- Parts printed at high enclosure temperatures exhibited greater strength than parts printed without the active addition of heat via the heater due to improved bond formation between individual layers of the print and a large degree of crystallinity through maintenance at these elevated temperatures.
- The FGM capability of this machine was proven and achieved through the control of temperature within the build environment, as demonstrated. Further refinement is needed to that ensure low crystalline printing does not come at the expense of inter-laminar strength.
- Since the current design allows for the movement of the x and y axes, it is not practically possible to enclose the base of the heated chamber. A method of relocating the movement of the x- or y-axis to be combined with the *z*-axis would allow the base of the machine to be sealed, thus allowing for a more controlled ambient temperature to form higher-quality parts. An independent system for actively cooling the print was identified as a requirement.
- The SEM testing results verified that FGM can take place without porosity or interlayer non-adhesion defects.

In this study, a low-cost FGM MEX 3D printer capable of printing high-quality PEEK parts was developed for under EUR 1000. This is significantly less than what is currently on the market This research contributes to expanding the possibilities of polymer utilisation, opening avenues for innovative material properties and advancing additive manufacturing techniques with high engineering grade polymers.

## Figures and Tables

**Table 1:** Physical properties of the purchased PEEK (manufacture datasheet).

Description	Value	Test Method
Density	1.31 g/cm^3^	ISO 1183
Tensile Modulus	3.8 GPa	ISO 527
Tensile Strength	97 MPa	ISO 527
Impact strength Notched Izod	7 Kj/m^2^	ISO1791eA

**Table 2:** Print parameters used to print test specimen.

Bed Preparation	Cleaned after Each Use, and a Thin Layer of Pritt Stick Was Applied
Adhesion Type	Brim (12 mm)
Print Speed	20 mm/s
Outer Perimeter Speed	10 mm/s
Inner Perimeter Speed	10 mm/s
Infill Speed	20 mm/s
Infill Pattern	±45°
Infill	100%
Flow Rate	75%
Extruder Temperature	390 °C
Bed Temperature	120–140 °C

**Table 3:** PEEK sample print parameters for first round of testing.

Sample	Extruder T °C	Bed T °C	Enclosure T (°C)	Bulkhead	Air Flow	Measured UTS (MPa)
A1	370	120	55	No	65%	24.08
A2	380	125	55	No	70%	29.85
A3	380	125	55	No	80%	32.70
A4	370	120	55	Yes	80%	31.97
A5	380	120	55	Yes	80%	36.44
A6	390	125	55	Yes	80%	46.65
B1	390	135	120	Yes	80%	49.87
B2	390	130	120	Yes	75%	43.39
B3	390	150	120	Yes	75%	47.57
B4	390	125	120	Yes	75%	43.67
B5	390	140	120	Yes	75%	47.44
B6	390	125	120	Yes	75%	47.10
C1	390	125	140	Yes	75%	30.07
C2	390	130	140	Yes	75%	47.07
C3	390	140	140	Yes	75%	32.09
C4	390	145	140	Yes	75%	48.40

**Table 4:** Thermal processing conditions for the properties of the two models.

	Test 1	Test 2
Bed temperature	120	140
Ambient air temperature	50	140
Radiation temperature	N/A	150
Nozzle temperature	390	390

## Data Availability

Publicly available testing data are available in this publication. Additional testing data may be granted through contact with the corresponding author.
